# Outcome of exercise-related out-of-hospital cardiac arrest is dependent on location: Sports arenas vs outside of arenas

**DOI:** 10.1371/journal.pone.0211723

**Published:** 2019-02-01

**Authors:** Matilda Frisk Torell, Anneli Strömsöe, Johan Herlitz, Andreas Claesson, Leif Svensson, Mats Börjesson

**Affiliations:** 1 Institution of Neuroscience and physiology, Gothenburg University, Gothenburg, Sweden; 2 County Council of Dalarna, Dalarna University, Falun, Sweden; 3 Prehospen Centre of Preshospital Research, Faculty of Caring Science, Work Life and Social Welfare, Borås, Sweden; 4 Center of Resuscitation and Science, Karolinska Institute, Stockholm, Sweden; 5 Institution of Neuroscience and physiology, Institution of Nutrition and Sport Science, University of Gothenburg, Sahlgrenska University Hospital/Östra, Gothenburg, Sweden; IRCCS Policlinico S.Donato, ITALY

## Abstract

**Background:**

The chance of surviving an out-of-hospital cardiac arrest (OHCA) seems to be increased if the cardiac arrests occurs in relation to exercise. Hypothetically, an exercise-related OHCA at a sports arena would have an even better prognosis, because of an increased likelihood of bystander cardiopulmonary resuscitation (CPR) and higher availability of automated external defibrillators (AEDs). The purpose of the study was to compare survival rates between exercise-related OHCA at sports arenas versus outside of sports arenas.

**Methods:**

Data from all treated exercise-related OHCA outside home reported to the Swedish Register of Cardiopulmonary Resuscitation (SRCR) from 2011 to 2014 in 10 counties of Sweden was analyzed (population 6 million). The registry has in those counties a coverage of almost 100% of all OHCAs.

**Results:**

3714 cases of OHCA outside of home were found. Amongst them, 268(7%) were exercise-related and 164 (61.2%) of those occurred at sports arenas. The 30-day survival rate was higher for exercise-related OHCA at sports arenas compared to outside (55.7% vs 30.0%, p<0.0001). OHCA-victims at sports arenas were younger (mean age±SD 57.6±16.3 years compared to 60.9±17.0 years, p = 0.05), less likely female (4.3% vs 12.2%, p = 0.02) and had a higher frequency of shockable rhythm (73.0% vs 54.3%, p = 0.004). OHCAs at arenas were more often witnessed (83.9% vs 68.9%, p = 0.007), received bystander CPR to a higher extent (90.0% vs 56.8%, p<0.0001) and the AED-use before EMS-arrival was also higher in this group (29.8% vs 11.1%, p = 0.009).

**Conclusion:**

The prognosis is markedly better for exercise-related OHCA occurring at sports arenas compared to outside. Victims of exercise-related OHCA at sports arenas are more likely to receive bystander CPR and to be connected to a public AED. These findings support an increased use of public AEDs and implementation of Medical Action Plans (MAP), to possibly increase survival of exercise-related OHCA even further.

## Introduction

Physical activity has long-term health benefits on cardiovascular health, including reduced mortality in cardiovascular disease. [1–4] Consequently, major international organizations are recommending regular exercise of moderate to vigorous intensity for health. [5–6] Recently, more studies are showing a U-shaped or J-shaped association with increased physical activity and cardiovascular health. [7–8] In case of an underlying heart disease (diagnosed or occult) the risk of a sudden cardiac arrest (SCA) is increased during and up to one hour after cessation of strenuous exercise, while regular activity seems to reduce the risk associated with vigorous activity bouts. [1,9–10]

The chance of surviving an out-of-hospital cardiac arrest (OHCA) has increased over the years due to improvements in several links in the classical “chain of survival”, although survival rates are still relatively low (2–20%). [11–12] Specifically, prior studies have shown that the chance of survival is higher if the cardiac arrest takes place in relation to exercise (as high as 50%). [13–16] Early bystander cardiopulmonary resuscitation (CPR) and early defibrillation are major determinants for survival from SCA and such favourable circumstances could at least partly explain the higher survival rates among exercise-related OHCA, compared to non-exercise related. [13–14,17–18]

Hypothetically, an exercise-related cardiac arrest occurring at a sports arena would have an even better prognosis in comparison to an exercise-related arrest occurring outside of such facility, since the likelihood of bystander CPR and availability of an automated external defibrillator (AED) is presumably even higher in this setting. A previous French study in fact reported higher survival rates in SCAs at sports arenas compared to those occurring outside of sports facilities. Interestingly, this was found despite a very low frequency of AED use (<1%). [19] Further studies are needed to determine whether these findings are applicable to other countries and in environments with a higher availability of AEDs.

Patients suffering exercise-related SCAs are in general younger (on average 10 years younger) compared to victims of non-exercise-related cardiac arrests, implying a lesser degree of coronary artery disease (CAD) in exercise-related SCA-victims. Still, the significant difference in survival rate remains after adjusting for age. [13–14] Paradoxically, the prognosis of exercise-related SCA in the age group < 35 years has been proposed to be worse compared to older age groups in some studies, [13,18] but more recent data seems to contradict these earlier findings. [20] Thus the role of age in survival of exercise-related SCA needs to be more elucidated.

The primary objective of the present study was to compare survival rates between exercise-related sudden cardiac arrests occurring at sports arenas versus outside of sports arenas, to better understand the higher survival rates in exercise-related OHCA. We also aimed to study whether there are other factors than exercise contributing to the high survival rate of exercise-related OHCA.

## Methods

This is a retrospective study from the Swedish Registry of Cardiopulmonary Resuscitation (SRCR), a registry which has previously been described in more detail. [14] All Emergency Medical Services (EMS) stations in Sweden participate and report to the registry, resulting in almost a 100% coverage of all OHCAs where CPR has been attempted. [21] The procedure of reporting an OHCA is performed in two steps. The EMS crew fills in the first part of a form with information from the EMS medical record. The second part is completed by a local CPR coordinator having access to in-hospital medical records. A complete form includes information regarding etiology, treatment and outcome of the OHCA. Etiology of the OHCA is defined by the EMS crew as being of “cardiac origin” or “other causes”. Outcome was defined as 30-day survival.

The study period reached from 1 January 2011 to 31 December 2014 and the study population consisted of inhabitants of ten Swedish counties. In total, the population was 6,092,861 in the year 2011 compared with 6,289,418 in the year 2014, representing 65% of Sweden’s population. [22]

Ethical permission was authorised by the ethical committee in Stockholm (Reference number 2015/1122-31/5).

All cases of OHCA occurring outside of home in the ten counties included in the study, were identified using the SRCR. Out of those OHCAs, a selection was made due to whether the cardiac arrest was exercise-related or not. This selection was made by manually search of the report sheet from EMS crew and CPR coordinators as well as EMS medical records.

Exercise-related SCA/SCD was defined as a cardiac arrest occurring during or within one hour after exercise, according to international standards, [13, 15, 20, 23] taken from EMS/in-hospital medical records. There was no differentiation regarding the intensity of physical activity. We chose to define a sports arena as a public location used for performance of physical activity, recreational or competitive, thereby including gyms/fitness centers as well as soccer stadiums for example.

Data variables collected from the SRCR and from EMS journals, for each case of exercise-related OHCA were reported in regards to UTSTEIN style [24] and included demographics, location (specifically whether the arrest took place in a sports arena or not), data regarding sports activities (type of sports and whether it occurred during a competitive event), circumstances of collapse[bystander witnessed, bystander CPR, public use of AED, initial rhythm (defined as shockable or non-shockable), CPR or AED use performed by First-responders, EMS response time and outcome [survival at 30 days].

A comparative statistical analysis was made between

1. Out of Hospital Cardiac Arrests that took place outside home and were related to exercise and cases that took place outside home and were not related to exercise.
  Here, the study population was also further subdivided according to age and sex and separate analyses were made among patients 0–35 years, 36–65 years and > 65 years.
2. Exercise-related OHCAs occurring at sports arenas and the exercise-related OHCAs occurring outside of sports arenas.

Descriptive statistics was used, and variables are presented as numbers, percentages, mean and median. In the comparison of proportions, Fishers Exact Test was used. In the comparison of continuous variables, Wilcoxons two-sample test was used. Differences in age adjusted survival was estimated with logistic regression and described as Odds Ratio (OR) with 95% Confidence Interval (CI). A p-value <0.05 was regarded as significant. Two sided tests were applied.

## Results

During the study period a total number of 3714 OHCAs occurring outside of home and where resuscitation was attempted were identified. Of these, 268 (7%) cases were exercise-related. The 30-day survival rate of exercise-related OHCA was 46.3% compared to 17.5% in the non-exercise-related group(p<0.0001). The age adjusted OR and 95% CI for survival to 30 days was 3.77;2.88–4.92; p<0.0001 when comparing exercise related OHCA with non-exercise related OHCA.

Characteristics of the exercise-related OHCAs according to their site of occurrence are presented in Table 1. Of the exercise-related OHCAs, 164(61.2%) occurred at sports arenas and 98(36.6%) occurred outside of such facilities (information of place of occurrence was missing in six cases). Patients presenting with OHCA at sports arenas were younger (mean age±SD 57.6±16.3 years compared to 60.9±17.0 years, p = 0.05). Overall there was a male predominance in both groups but victims at sports arenas were even less likely to be female (4.3% vs 12.2%, p = 0.02).

**Table 1:** Characteristics and outcome in relation to physical activity at a sport arena or not.

	OHCA at a sport arena		p**
	Yes	No	
	N = 164	N = 98	
**Age (years)** (5, 5) *	57.6 ±16.3	60.9 ±17.0	0.05
**Sex** (0, 0)			
Women	7/164 (4.3)	12/98 (12.2)	0.02
**Initial arrhythmia** (12, 4)			
Ventricular fibrillation	111/152 (73.0)	51/94 (54.3)	0.004
**Etiology** (16, 5)			
Cardiac	116/148 (78.4)	70/93 (75.3)	0.64
**Witnessed status**			
Crew witnessed (3, 8)	9/161 (5.6)	11/90 (12.2)	0.09
Bystander witnessed (3, 8)	135/161 (83.8)	62/90 (68.9)	0.007
Non-witnessed (3, 7)	17/161 (10.6)	17/91 (18.7)	0.08
**Early CPR**			
CPR before dispatched unit (4, 3)			
Yes	144/160 (90.0)	54/95 (56.8)	<0.0001
If yes, was a defibrillator attached? (3, 0)			
Yes	42/141 (29.8)	6/54 (11.1)	0.009
If yes, was the patient defibrillated before arrival of dispatched unit? (1, 0)			
Yes	32/41 (78.0)	5/6 (83.3)	1.00
CPR by fire brigade/police before EMS arrival?			
Yes (2, 2)	22/162 (13.6)	17/96 (17.7)	0.37
If yes, was a defibrillator attached?			
Yes (2, 0)	15/20 (75.0)	11/17 (64.7)	0.72
If yes, was the patient defibrillated before EMS arrival?			
Yes (2, 4)	11/13 (84.6)	4/7 (57.1)	0.29
**Delay time** (median, min)			
From collapse–Call (24, 20)	2	2	0.82
From collapse–CPR (13, 6)	1	3	<0.0001
From collapse–defibrillation (25, 211)	10	12	0.03
From dispatch–arrival on scene (38, 26)	8	11	0.19
**Outcome**			
ROSC at any time			
Yes (3, 4)	100/161 (62.1)	44/94 (46.8)	0.02
ROSC on arrival in hospital			
Yes (5, 3)	95/159 (59.8)	41/95 (43.2)	0.01
Alive at 30 days			
Yes (5, 5)	88/158 (55.7)	28/92 (30.4)	<0.0001

* Number of patients with missing information

** p-value

CPR, cardiopulmonary resuscitation; EMS, emergency medical service; ROSC, return of spontaneous circulation.

Out-of-hospital cardiac arrests occurring at sports facilities were more likely to be bystander witnessed (83.9% vs 68.9%, p = 0.007) and were also more frequently receiving CPR before arrival of EMS (90.0% vs 56.8%, p<0.0001). AED-connection prior to EMS-arrival was more likely to occur when the OHCA took place at a sports arena compared to outside (29.8% vs 11.1%, p = 0.009). Initial rhythm differed significantly between the groups, with a higher frequency of shockable rhythm i.e. ventricular tachycardia or ventricular fibrillation for cardiac arrests at sports arenas (73.0% vs 54.3%, p = 0.004).

Delay time from collapse to call for EMS did not differ between the groups (median 2.0 min, p = 0.82) nor was there a significant difference in the median delay time from call to arrival of EMS (8min vs 11min, p = 0.19). The median delay time from collapse to start of CPR (1 min vs 3 min, p<0.0001) and from collapse to defibrillation (10min vs 12.5 min, p = 0.03) differed in favor of occurrence at sports facilities.

Survival at 30 days was higher among exercise-related OHCA occurring at sports arenas compared to those occurring outside (55.7% vs 30.0%, p<0.0001). The age adjusted OR and 95% CI was 2.85; 1.64–5.02; p = 0.0002 when exercise-related OHCA at sports arena were compared with those occurring outside. Return of spontaneous circulation at any time was more common in the sports arena group (62.1% vs 46.8%, p = 0.02) and patients in this group more often had spontaneous circulation at hospital admission (59.8% vs 43.2%, p = 0.01).

Victims in both groups were considered to have an OHCA of cardiac origin to the same extent (78.4% vs 75.3%, p = 0.64).

The higher survival rates among exercise-related OHCA remained significant throughout the study population when comparing different age-groups. There were in total 22 cases and 13 survivors of exercise-related OHCA in the age-group 0–35 years and a survival rate of 59.1% compared to 16.4% in the non-exercise-related group, which included 214 cases and 35 survivors(p<0.0001). In the age group 36–65 years, there were 68 survivors out of 136 cases in the exercise-related group and 239 survivors out of 1096 in the non-exercise-related group, with a survival rate of 50% vs 21.8%(p<0.0001). Among victims older than 65 years, 36 out of 90 survived their exercise-related OHCA compared to 293 out of the 1812 who suffered a non-exercise-related OHCA, resulting in a difference in survival rate of 40.0% vs 16.2%(p<0.0001). Survival rates in relation to exercise, gender and age are presented in Table 2.

**Table 2:** Survival to 30 days.

	Physical activity		p***
	Yes	No	
**All patients** (11, 130) *	118/255 (46.3) **	579/3316 (17.5)	<0.0001
**Sex**			
Men (11, 111)	113/235 (48.1)	454/2430 (18.7)	<0.0001
Women (0, 18)	5/20 (25.0)	125/886 (14.1)	0.19
**Age** (years)			
0–65 (4, 27)	81/158 (51.3)	274/1310 (20.9)	<0.0001
0–35 (1, 8)	13/22 (59.1)	35/214 (16.4)	<0.0001
36–65 (3, 19)	68/136 (50.0)	239/1096 (21.8)	<0.0001
> 65 (3, 10)	36/90 (40.0)	293/1812 (16.2)	<0.0001

* Number of patients with missing information

** Number of survivors/ Number of patients evaluated (percentage of survivors)

*** p-value

Male victims suffering OHCA in relation to exercise had significantly higher survival rates in comparison to male victims of non-exercise-related OHCA (48% vs 18.7%, p<0.0001). On the other hand, there was no statistically significant difference in survival rates comparing female victims of exercise-related OHCA with females suffering non-exercise-related cardiac arrest (25.0% vs 14.1%, p = 0,19). However, the number of females with OHCA was much lower.

**Fig 1:**
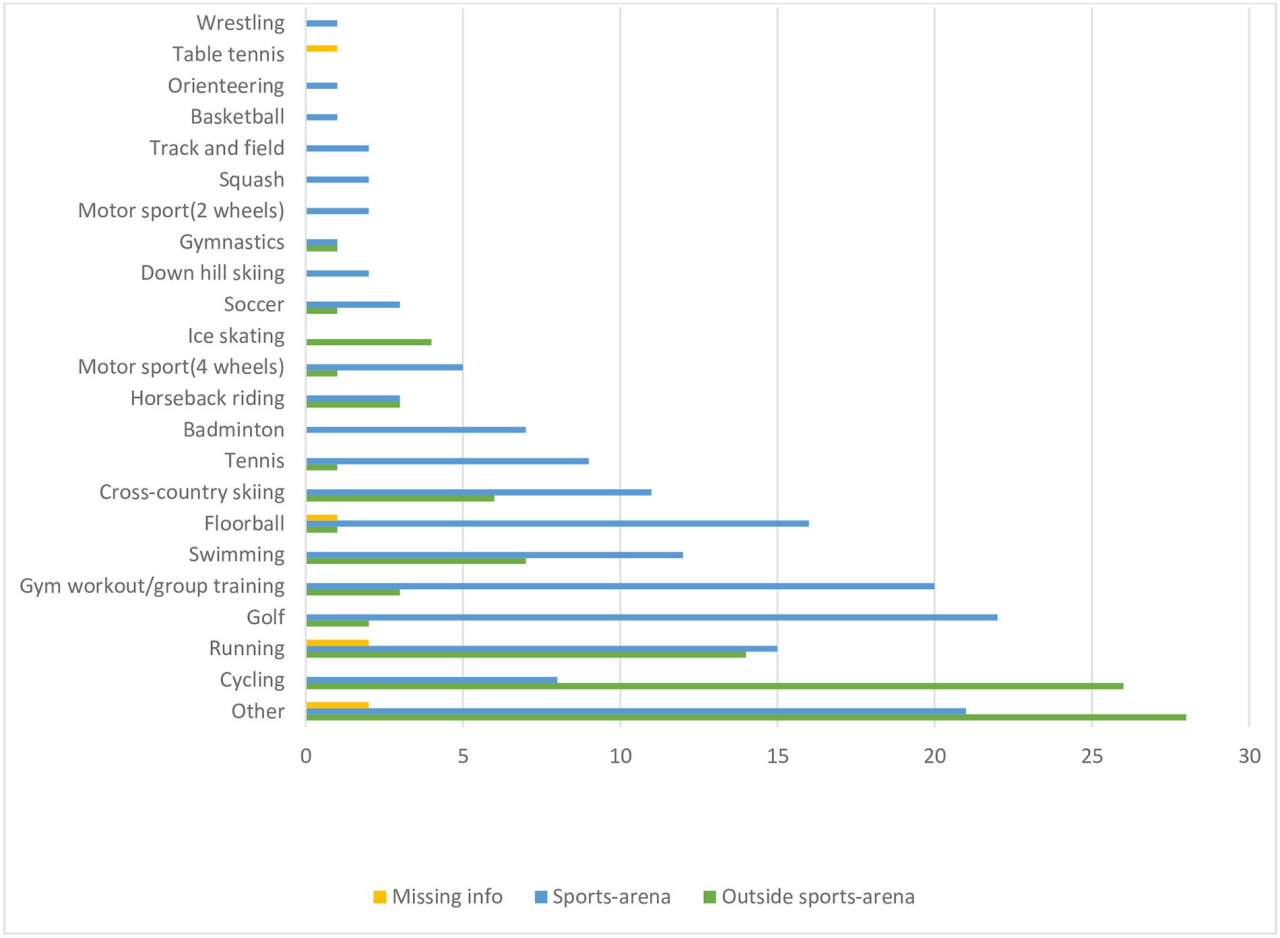
Number of cardiac arrests in relation to sport and location.

The most common sport activity associated with exercise-related OHCA was cycling (*n* = 34, 13%), followed by running (*n* = 31, 12%), golf (*n* = 24, 9%) and gym workout/group training (*n* = 23, 9%). Number of cardiac arrests in relation to sport activity and location is presented in Fig 1.

## Discussion

The main finding of this retrospective study from the Swedish registry of cardiopulmonary resuscitation is that exercise-related OHCAs at sports arenas have a better prognosis than exercise-related OHCAs outside of sports arenas. In addition, we could confirm a higher survival rate for exercise-related OHCA overall, consistent with previous studies. [13–15]

The 30-day survival rate of 55.7% among OHCAs at sports arenas is higher compared to a previous similar French study by Marijon, which reported a survival rate of 22.8% at sports facilities compared to 8.0% among exercise-related OHCAs occurring outside. Witness degree, bystander CPR and initial shockable rhythm, correlate to better prognosis in both the French and the present study. A possible explanation for the difference in survival rates is the frequency of AED use, since there was a remarkably low AED use (<1%) in the French study compared to the present Swedish study, which report an AED use of 29.8% in sports facilities. Another difference was the lower frequency of bystander CPR in the French study (35.4% at arenas), despite a very high witness degree (99.8%). [19] Several studies have shown that patients suffering an exercise-related SCA are more likely to have an initial shockable rhythm. [13–14, 25] In the present study, 73% of patients suffering an exercise-related OHCA at a sports arena had a shockable rhythm, thus these patients would most likely take great benefit from early defibrillation. When considering the exercise-related OHCAs occurring outside of sport arenas, the median delay time from collapse to EMS arrival and defibrillation was longer and the cardiac arrest thereby had been ongoing for a longer time period, with a lower frequency of bystander CPR. These circumstances could of course explain why these cases were less likely to have a shockable rhythm.

The highest survival rate was found in the age group 0–35 years (59.1%), not supporting some studies showing a lower survival rate in the young, athletic SCA-patients [13, 18] The reason proposed has been that the etiology of the younger athletes is different (more commonly congenital and/or inherited disease) compared to underlying CAD in the older SCA-victims. More recent studies have not shown such figures, possibly due to less selection bias. [20, 26] These results are clinically important as the underlying diseases are different in the young compared to older athletes. In the latter group, the cause of SCA is mainly CAD. [13, 15, 27–28] Preventive measures thus may have effect also in young athletes. However, the low number of young SCA- victims in the present study, indicates the need for further studies investigating the role of age in survival from exercise-related SCA.

A surprise finding was that, while females have a lower incidence of exercise-related OHCA overall, compared to men, [13–14, 18, 27–28] this was even more pronounced in sports arenas (only 4% of victims were female). Besides the proposed lower participation rate of women in intense sport activities worldwide, other explanations for the lower incidence of exercise-related OHCA in women, include the relatively lower prevalence of CAD in women at certain age (before menopause) and small differences in underlying cardiovascular disease patterns. [1, 15, 29] However, the survival rate in case of exercise-related OHCA, seems to be lower than for men (25% vs 48.1%). Important to note here is the overall lower incidence of SCA in women and that SCA among women is less likely to occur at public locations which also correlates with a lower frequency of VF and thereby worse prognosis. [30–31] In other words, these findings are most likely not isolated to exercise-related SCA.

The present study has several important clinical implications. Previous studies have demonstrated the benefits of public CPR and AEDs. For example, the use of AEDs has resulted in higher survival rates in US High Schools, [32–33] airports, on airplanes and in casinos. [34–36] Nonetheless, AED availability itself does not guarantee successful defibrillation, since the device also must be properly used. Education in AED use among lay persons and the use of structured AED programs have been shown to significantly increase the number of survivors of OHCA in public locations. [37–39] At sports arenas, AEDs are preferably parts of a structured medical action plan (MAP) in order to increase the likelihood of coordinated and safely executed care in the event of cardiac arrests as well as other medical emergencies. [40–42] Yet, as the availability of AEDs and the existence of MAPs at sports arenas are still insufficient, studies such as the present is very important. In Sweden, there are approximately 15 000 AEDs registered, most of them could be found at public locations, however we do not know to what extent sports arenas are included. [21] The increased prevalence of defibrillators in sports arenas and the increased survival figures compared to earlier studies, emphasize the potential importance of implementing existing recommendations for arena safety to all arenas. Furthermore, as the survival is higher at sports arenas, associated to a higher AED use and witness density, similar recommendations outside of sports arenas (during sports events such as cycling or running competitions but also in other public locations) would possible increase OHCA survival rates overall. Thus, the findings in the present study support an extension of number of AEDs placed in sports facilities and at other strategic public places as well as the education and regular training on how to use them.

The present study has its limitations. The low number of cases in the youngest age group (0–35 years) as well as the low number of female victims make it difficult to draw firm conclusions about survival in these groups. Although the SRCR covers nearly 100% of all OHCAs were resuscitation has been attempted, there is still a risk that some unwitnessed exercise-related OHCA are not reported or wrongly classified as non-exercise-related. Furthermore, in some non-witnessed cases there is a possibility that CPR was never attempted. We do lack full information about the underlying diagnosis of the OHCAs and the assessment whether the OHCA is of cardiac origin or not is made by EMS crew, which is a potential limitation. Finally, the study cohort includes patients up to the end of 2014 only. However, during the last three years the overall 30-day survival rate of OHCA has remained unchanged. [21]

In conclusion, survival rates are markedly better in exercise-related OHCA occurring at sports arenas vs outside arenas. A significantly higher degree of witnessed arrests and AED use at sports arenas are most likely of great importance. Our results emphasize a more widespread use of AEDs and the implementation of a structured emergency preparedness as part of MAPs, not just at sports arenas but also in other public locations where people engage in exercise. Interestingly, the survival rate was higher for exercise-related OHCA at all ages, while there was a tendency towards worse prognosis for females, encouraging further studies on mechanisms of exercise-related OHCA in different age groups and among females.
